# Sustainability of community-led total sanitation outcomes: Evidence from Ethiopia and Ghana

**DOI:** 10.1016/j.ijheh.2017.02.011

**Published:** 2017-05

**Authors:** Jonny Crocker, Darren Saywell, Jamie Bartram

**Affiliations:** a The Water Institute, University of North Carolina at Chapel Hill, USA; b Plan International USA, USA

**Keywords:** CLTS, Sustainability, Sanitation, Hygiene, Ethiopia, Ghana

## Abstract

We conducted a study to evaluate the sustainability of community-led total sanitation (CLTS) outcomes in Ethiopia and Ghana. Plan International, with local actors, implemented four CLTS interventions from 2012 to 2014: health extension worker-facilitated CLTS and teacher-facilitated CLTS in Ethiopia, and NGO-facilitated CLTS with and without training for natural leaders in Ghana. We previously evaluated these interventions using survey data collected immediately after implementation ended, and concluded that in Ethiopia health extension workers were more effective facilitators than teachers, and that in Ghana training natural leaders improved CLTS outcomes. For this study, we resurveyed 3831 households one year after implementation ended, and analyzed latrine use and quality to assess post-intervention changes in sanitation outcomes, to determine if our original conclusions were robust. In one of four interventions evaluated (health extension worker-facilitated CLTS in Ethiopia), there was an 8 percentage point increase in open defecation in the year after implementation ended, challenging our prior conclusion on their effectiveness. For the other three interventions, the initial decreases in open defecation of 8–24 percentage points were sustained, with no significant changes occurring in the year after implementation. On average, latrines in Ethiopia were lower quality than those in Ghana. In the year following implementation, forty-five percent of households in Ethiopia repaired or rebuilt latrines that had become unusable, while only 6% did in Ghana possibly due to higher latrine quality. Across all four interventions and three survey rounds, most latrines remained unimproved. Regardless of the intervention, households in villages higher latrine use were more likely to have sustained latrine use, which together with the high latrine repair rates indicates a potential social norm. There are few studies that revisit villages after an initial evaluation to assess sustainability of sanitation outcomes. This study provides new evidence that CLTS outcomes can be sustained in the presence of training provided to local actors, and strengthens previous recommendations that CLTS is not appropriate in all settings and should be combined with efforts to address barriers households face to building higher quality latrines.

## 1 Introduction

Globally, 2.4 billion people lack improved sanitation, and 946 million people practice open defecation (WHO/UNICEF, 2015). The United Nations reaffirmed the importance of sanitation by including it in the Sustainable Development Goals (SDGs), which calls for ending open defecation and universal access to adequate and equitable sanitation (UN General Assembly, 2015). The SDGs also set out the means of implementation as strengthening the participation of local communities and capacity building support for developing countries. Community-led total sanitation (CLTS) is an approach to addressing open defecation that triggers emotions to generate a collective demand for sanitation within a community. CLTS emerged in the year 2000, and has since spread to over 60 countries, many of which now include it in national policy (Institute of Development Studies, 2016). CLTS has a role to play in addressing the SDGs, as it is participatory, generally includes capacity building, and has shown promise in addressing open defecation (Kar and Chambers, 2008, Pickering et al., 2015). However, it is not always effective (Guiteras et al., 2015), and seems to be most appropriate under certain settings, such as high baseline open defecation (Crocker et al., 2016b) and high social capital (Cameron et al., 2015, Crocker et al., 2016a).

There are no journal-published studies on the sustainability of CLTS outcomes. Three gray literature studies (literature not published in scientific journals) report sanitation outcomes and rates of reversion back to open defecation 2–4 years after CLTS completed (Hanchett et al., 2011, Mukherjee et al., 2012, Tyndale-Biscoe et al., 2013). Another report reviewed gray literature on CLTS sustainability, and while it described a number of methodological challenges to drawing any conclusions across the varied reports, it does include a thorough discussion of factors that enable and constrain the sustainability of CLTS outcomes (Cavill et al., 2014). The review found that CLTS outcomes were reported to be more sustainable where there was a supportive enabling environment (e.g. sufficient follow-up visits were conducted), where communities had market-access to latrine products and materials, and where communities were socially cohesive.

There are very few studies that report on the sustainability of any type of sanitation intervention (Garn et al., 2016), and generating evidence on longer term outcomes of sanitation interventions is a research priority (Waddington et al., 2009). Two studies report longer term latrine use following sanitation interventions: one 5 years after a latrine-provision project in Bangladesh (Hoque et al., 1996), and another 2–9 years after programs across 8 countries that included latrine promotion (Cairncross and Shordt, 2004).

We conducted a study to assess how sanitation outcomes of four CLTS interventions in Ethiopia and Ghana changed one year after the interventions had finished. We previously published evaluations of the four CLTS interventions that were based on surveys conducted before and immediately after the interventions (Crocker et al., 2016a, Crocker et al., 2016b). The previously published evaluations focus on the effectiveness of training health workers, teachers, and natural leaders to lead or support CLTS facilitation, and include recommendations on where and how to engage these local actors. In the initial evaluations, we found that open defecation decreased during all four interventions. In Ethiopia, teacher-facilitated CLTS was initially less effective than health extension worker-facilitated CLTS. In Ghana, training natural leaders increased the impact of CLTS. Thus, our second objective in this study was to assess if the conclusions from the original evaluations are still sound given new longer-term survey data. Our third objective was to assess other predictors of sustained latrine use.

## 2 Methods

### 2.1 Program description

Four different CLTS interventions were implemented: in Ethiopia, (1) health extension worker (HEW) and kebele leader-facilitated CLTS, and (2) teacher-facilitated CLTS; and in Ghana, (3) NGO-facilitated CLTS, and (4) NGO-facilitated CLTS, with additional training for natural leaders. A kebele is the lowest administrative unit in Ethiopia, comprising 20–30 villages and approximately 5000 people in rural areas. Kebele leaders and HEWs always worked together in intervention 1, so the impact of each of these actors cannot be separated. Natural leaders are motivated community members who encourage others to construct latrines and change sanitation-related behaviors. Facilitation comprised visits to study villages by facilitators to conduct the three typical stages of CLTS as they are described in the CLTS Handbook (Kar and Chambers, 2008): pre-triggering (or community entry), triggering, and follow-up, which involves monitoring a community's progress and guiding them toward eliminating open defecation. The two interventions in Ethiopia lasted 12 months, and the two in Ghana lasted 18 months. Interventions 1 and 2 in Ethiopia began with training local actors who then led CLTS facilitation. Interventions 3 and 4 in Ghana were facilitated by Plan. Intervention 4 included the addition of training natural leaders after triggering had been completed so they could support facilitation. These four CLTS interventions cover a range of implementation arrangements and modalities as practiced by other organizations and in other countries (Venkataramanan, 2016, Venkataramanan, 2012), so the findings are relevant beyond this project. A timeline of implementation activities and ODF certification rates are in the appendix (Tables S1–S3). Detailed implementation narratives are available online (Plan International Ethiopia, 2015, Plan International Ghana, 2015).

There were a range of pre-existing factors that enabled the CLTS interventions, and could contribute to sustainability. In both Ethiopia and Ghana there were supportive national governments that have produced policies or strategies naming CLTS as the preferred rural sanitation approach, national guidelines for CLTS implementation, and CLTS coordinating committees. Moreover, local government is mandated with implementing CLTS (Crocker and Bogle, 2015, Crocker and Rowe, 2015). Plan spent the year preceding the interventions working with government and NGO partners to develop contextually appropriate training manuals that would be used for the interventions (Plan International Ethiopia, 2012, Plan International Ghana, 2013).

### 2.2 Study design

The study in Ethiopia used a quasi-experimental design, in which kebeles (clusters of villages) were prematched on latrine access and population, then manually assigned to receive CLTS facilitated by either HEWs and kebele leaders, or by teachers. The study in Ghana used a cluster-randomized design, in which all project villages received CLTS, and half of the villages were randomly selected to receive natural leader training as an add-on activity. The interventions in Ethiopia took place in the Oromia and Southern Nations, Nationalities, and Peoples (SNNP) regions, and in Ghana in the Central, Upper West, and Volta regions. Further details on the two study designs are in the previous publications.

In Ethiopia, a complete village listing was conducted at baseline, then villages were randomly sampled and all households within sampled villages were surveyed (Table S4 in the appendix). The same households were resurveyed immediately after the interventions (midline) and again one year later (endline). In Ghana, a complete household listing was conducted immediately after the interventions, then households were randomly sampled, surveyed for the midline, and resurveyed a year later for the endline. No baseline survey was used in Ghana.

Household surveys covered demographics, sanitation, hygiene, interactions, and recall of CLTS events. Sanitation outcomes were assessed by asking heads-of-households where members of their family primarily defecated and their handwashing practices. Those reporting using a latrine were asked a series of questions to determine if it was private, shared, or communal. Latrine and handwashing station quality and maintenance were then assessed by observation. All data collection was conducted by an independent contractor in each country. Surveys were translated into local languages then independently checked, pretested during surveyor training, and piloted in non-study villages. Surveyors were audited by Plan staff or team leaders resurveying a selection of households. Printed surveys were used in Ethiopia, and SurveyCTO software on Nexus tablets was used in Ghana.

The primary outcome was change in levels of open defecation at the household-level. Open defecation was defined as respondents reporting their family's primary place of defecation as somewhere other than a latrine. Additionally, if a respondent reported using a private latrine but did not allow the surveyor to observe it, their household was categorized as open defecation, as were households whose latrines were observed to be full or have collapsed floors. Baseline surveys were not used in Ghana, so baseline open defecation was estimated using the conservative assumption that decreases in open defecation were equivalent to increases in latrine ownership. Baseline latrine ownership in Ghana was estimated based on self-reported latrine age at the midline survey. Secondary outcomes assessed were latrine quality and condition, and access to handwashing materials. Multivariable logistic regression was used to analysis demographic and sanitation variables as potential predictors of sustained latrine use. Following rationale described in a handpump sustainability study, we ran one combined model per country rather than a stepwise model (Foster, 2013). All analysis was completed in STATA 13/SE. The “svyset” command was used to account for clustering of outcomes within villages, unequal selection probabilities, and nonresponse rates (“svyset village [pweight = weight], strata(region) || household”, in which sampling was stratified by region, villages were the primary sampling unit, households were the secondary sampling unit, and weights were calculated from selection probabilities and nonresponse rates).

This study was reviewed and approved by the Office of Human Research Ethics of the University of North Carolina, Chapel Hill (Ethiopia study #: 12-1851, Ghana study #: 12-1970). Local approval was obtained from zonal and district health offices in Ethiopia, and regional environmental health and sanitation directorates in Ghana. Informed consent was received from all respondents.

## 3 Results

The Ghana study population had more education, higher indicators of wealth (metal roofing, and owning a radio or television), and were more likely to use an improved water supply than the Ethiopia study population (Table 1). Baseline ownership of a private latrine was much higher in the Ethiopia study population. Within each country, the study population differed substantially between regions (Table 2).

**Table 1:** Household and respondent characteristics in villages receiving CLTS in Ethiopia and Ghana, by intervention.

Variable	Ethiopia			Ghana		
	HEW CLTS	Teacher CLTS	Difference [95% CI]	NGO CLTS	NGO CLTS + NL training	Difference [95% CI]
Female respondent	73%	77%	4% [−1%, 8%]	74%	69%	−5% [−13%, 2%]
Five or more years of education^a^	20%	17%	−3% [−8%, 2%]	52%	58%	7% [−8%, 22%]
Household size (people)	6.1	5.7	−0.4 [−0.6, −0.2]	4.1	3.9	−0.2 [−1, 0.5]
Number of children per household	0.9	0.9	0 [−0.1, 0.1]	0.7	0.6	−0.1 [−0.3, 0.1]
Metal roof	28%	19%	−10% [−15%, −4%]	88%	93%	5% [−4%, 14%]
Own radio	26%	27%	1% [−5%, 7%]	48%	50%	2% [−6%, 9%]
Own television	1%	1%	0% [−1%, 1%]	34%	41%	7% [−3%, 16%]
Years family lived in village	24	21	−2 [−4, −1]	31	25	−5 [−10, −0.3]
Years family lived in current house	15	13	−3 [−5, −1]	15	14	−2 [−5, 1]
Use improved water supply	51%	51%	0% [−15%, 14%]	77%	77%	0% [−10%, 11%]
Baseline latrine ownership	84%	76%	−8% [−12%, −3%]	9%	13%	3% [−1%, 8%]

*Abbreviations*: HEW, health extension worker; NGO, non-governmental organization; NL, natural leader.

a Assumes that respondents who have completed primary education in Ghana have spent at least five years in education. All analysis accounts for unequal selection probabilities, non-response rates, and village-clustering. All Ethiopia values are from the baseline survey. All Ghana values are taken from the midline survey, and describe the two treatment groups at that time, except for latrine ownership private latrine ownership, which is based on recall of how old their latrines were.

**Table 2:** Household and respondent characteristics in villages receiving CLTS in Ethiopia and Ghana, by region.

Variable	Ethiopia		Ghana^a^		
	Oromia	SNNP	Central	Upper West	Volta
Female respondent	74%	77%	68%	95%	62%
Five or more years of education^b^	13%	21%	57%	15%	75%
Household size	6.1	5.6	3.3	6.4	3.6
Number of children per household	1.0	0.9	0.5	1.1	0.5
Metal roof	11%	29%	97%	72%	94%
Own radio	30%	24%	43%	49%	55%
Own television	0%	1%	46%	19%	38%
Years family lived in village	25	21	27	36	25
Years family lived in current house	6	18	14	16	15
Use improved water supply	16%	75%	65%	41%	60%
Baseline latrine ownership	51%	98%	24%	10%	25%
Baseline open defecation^c^	70%	27%	34%^c^	96%^c^	36%^c^
Village size	29	38	164	68	123
Villages with prior WaSH projects (%)	0%	0%	100%	45%	79%
Villages with prior subsidized latrines (%)	0%	0%	33%	15%	37%

a All Ghana values are taken from the midline household census and survey, and describe the two treatment groups at that time, except for baseline private latrine ownership, which is based on recall of how old their latrines were.

b Assumes that respondents who have completed primary education in Ghana have spent at least five years in education.

c Baseline surveys were not used in Ghana, so baseline open defecation was based on the conservative assumption that decreases in open defecation were equivalent to increases in latrine ownership.

**Fig. 1:**
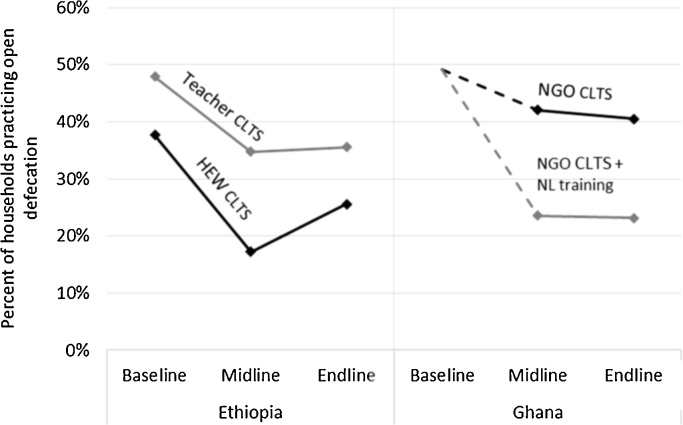
Open defecation in villages receiving four CLTS interventions in Ethiopia and Ghana. Abbreviations: HEW, health extension worker; NGO, non-governmental organization; NL, natural leader. Baseline was just before the interventions began. Midline was after CLTS interventions ended (12-months post-baseline in Ethiopia, and 18-months post-baseline in Ghana). Endline was 1-year after midline. Baseline surveys were not used in Ghana, so baseline open defecation was based on the conservative assumption that decreases in open defecation were equivalent to increases in latrine ownership.

*During* implementation (baseline to midline), open defecation decreased for all four CLTS interventions (Fig. 1 and Table 3). In the year *after* implementation (midline to endline), open defecation increased by 8 percentage points for the HEW-facilitated CLTS intervention, and did not change significantly for the other three interventions. HEW-facilitated CLTS was initially more effective than teacher-facilitated CLTS; however, by the endline both facilitation approaches in Ethiopia were associated with a net 12-percentage point decrease in open defecation. In Ghana, the initial impact of the natural leader training on open defecation was maintained in the year after the interventions stopped.

**Table 3:** Open defecation in villages receiving four CLTS interventions in Ethiopia and Ghana.

	Intervention	Baseline^a^	Midline^b^	Endline^c^	Baseline to midline	Midline to endline	Baseline to endline
Ethiopia	Full sample (both interventions)	45%	30%	31%	−15%	2%	−13%
		[42, 48]	[25, 34]	[26, 37]	[−20, −11]	[−3, 7]	[−19, −8]
	HEW CLTS	38%	17%	26%	−20%	8%	−12%
		[34, 42]	[14, 20]	[20, 31]	[−26, −15]	[3, 14]	[−20, −4]
	Teacher CLTS	48%	35%	36%	−13%	1%	−12%
		[44, 52]	[29, 41]	[28, 43]	[−19, −7]	[−7, 8]	[−20, −4]

Ghana	Full sample (both interventions)	49%^a^	33%	32%	−16%	−1%	−17%
		–	[25, 41]	[25, 39]	–	[−4, 2]	–
	NGO CLTS	49%^a^	42%	41%	−7%	−2%	−9%
		–	[28, 56]	[28, 53]	–	[−5, 2]	–
	NGO CLTS + NL training	49%^a^	24%	23%	−26%	0%	−26%
		–	[17, 30]	[17, 29]	–	[−5, 4]	–

*Abbreviations*: HEW, health extension worker; NGO, non-governmental organization; NL, natural leader.

a Baseline was just before the interventions began. Baseline surveys were not used in Ghana, so baseline open defecation was based on the conservative assumption that decreases in open defecation were equivalent to increases in latrine ownership.

b Midline was after CLTS interventions ended (12-months post-baseline in Ethiopia, and 18-months post-baseline in Ghana).

c Endline was 1-year after midline. 95% confidence intervals are in square brackets. All analysis accounts for unequal selection probabilities, non-response rates, and village-clustering.

**Fig. 2:**
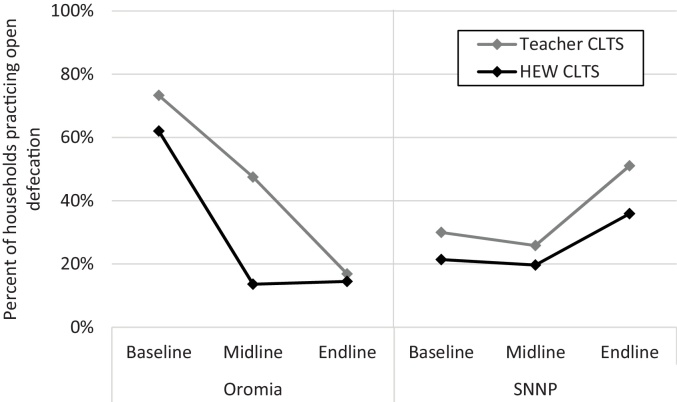
Open defecation in villages receiving CLTS interventions in Ethiopia, by region. Abbreviations: HEW, health extension worker; SNNP, Southern Nations, Nationalities, and Peoples. Baseline was before the interventions began. Midline was after CLTS interventions ended (12-months post-baseline in Ethiopia). Endline was 1-year after midline.

**Fig. 3:**
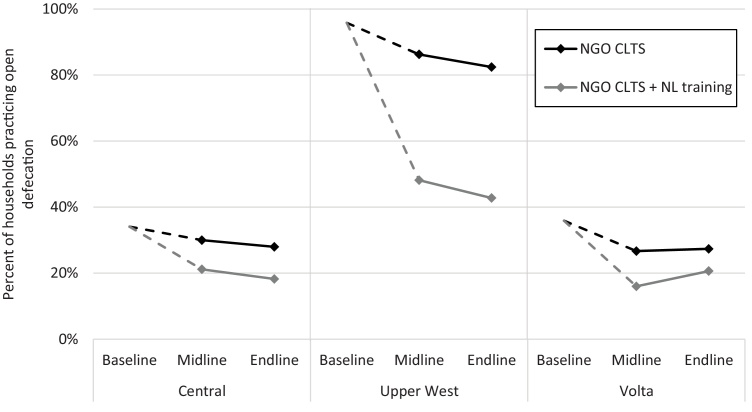
Open defecation in villages receiving CLTS interventions in Ghana, by region. Abbreviations: NGO, non-governmental organization; NL, natural leader. Baseline was before the interventions began. Midline was after CLTS interventions ended (18-months post-baseline in Ghana). Endline was 1-year after midline.

In the Oromia region in Ethiopia, teacher-facilitated CLTS was associated with a continued decrease in open defecation from midline to endline, “catching up” with HEW-facilitated CLTS (Fig. 2). However, both interventions in Ethiopia were associated with slight increases in open defecation in the SNNP region from midline to endline. Further analysis revealed that 3% of the latrines that were in use in Oromia at the midline were collapsed or otherwise unusable at the endline, while in the SNNP region 38% of latrines used at the midline were unusable at the endline. In Ghana, there were mostly no statistically significant changes in open defecation for either intervention in any region, with the exception of villages receiving natural leader training in the Volta region in which open defecation increased slightly (Fig. 3). Latrine use disaggregated by private, shared, and communal latrines is presented in the appendix (Fig. S1).

Ownership of a private latrine was much higher in the Ethiopia study villages than in Ghana at all survey-time points (Table 4). The latrines in Ghana were higher quality on average, with a greater proportion qualifying as improved, and a much greater proportion having an intact superstructure that provided privacy to the user. The decrease in open defecation during the interventions in Ethiopia corresponded to no change in household latrine ownership, but instead to an increase in the quality, condition, and use of latrines. The decrease in open defecation during the interventions in Ghana corresponded to an increase in household ownership of latrines, while the quality and condition of latrines remained relatively constant over time. There were no substantial changes in latrine quality in the year after the interventions. At all survey time-points, less than 25% of latrines had handwashing materials available. At the endline survey, 45% of households owning latrines in Ethiopia reported that they had to repair or rebuild their latrines in the preceding year, while only 5% did in Ghana (Table 4).

**Table 4:** Private latrine ownership and quality in villages receiving CLTS interventions in Ethiopia and Ghana.

Variable	Ethiopia			Ghana		
	Baseline	Midline	Endline	Baseline^b^	Midline	Endline
Self-reported latrine ownership	79%	79%	82%	15%	30%	35%
Proportion of private latrines						
Observed by surveyor	100%	98%	99%	97%	97%	90%
Improved^a^	22%	21%	22%	24%	27%	33%
Stable and safe flooring	68%	81%	76%	–	86%	80%
Complete privacy	5%	8%	8%	–	58%	61%
Clean (no feces on floor)	61%	68%	66%	–	80%	65%
Less than ∼10 flies	71%	79%	76%	–	70%	81%
With handwashing materials	17%	24%	18%	–	14%	13%
Requiring repair in previous year	–	–	45%	–	–	5.4%

a Based on the Joint Monitoring Program definition, though measurement of improved latrines varies globally (Bartram et al., 2014).

b Baseline surveys were not used in Ghana, so baseline latrine characteristics are based on self-reported latrine age, and latrine characteristics prone to changing are not reported.

Logistic regression was used to identify variables that could predict sustained latrine use (Table 5). There was only one consistent predictor of sustained latrine use across both Ethiopia and Ghana: households in villages with over 75% latrine use at the midline were more likely to sustain their own latrine use over the following year. In Ethiopia, households in the SNNP region were far less likely to sustain latrine use than those in the Oromia region, even when controlling for other variables (Table 5, Ethiopia adjusted model). Households with a metal roof (an economic indicator) were also more likely to sustain latrine use in Ethiopia, while households with access to an improved drinking water source were less likely to.

**Table 5:** Predictors of sustained latrine use in the year following CLTS interventions in Ethiopia and Ghana.

Explanatory variables	Ethiopia		Ghana	
	Unadjusted odds ratio	Adjusted odds ratio	Unadjusted odds ratio	Adjusted odds ratio
Teacher CLTS/NL training	0.69*	0.93	1.00	0.88
	[0.45, 1.06]	[0.53, 1.61]	[0.62, 1.63]	[0.51, 1.51]
Oromia/Central region	1	1	1	1
SNNP/Upper West region	0.19***	0.17***	0.50*	0.92
	**[0.12, 0.30]**	**[0.1, 0.29]**	**[0.24, 1.03]**	[0.34, 2.47]
–/Volta region			0.68*	0.92
			**[0.43, 1.08]**	[0.49, 1.71]
Household size	1.03	1.00	0.95	0.97
	[0.98, 1.08]	[0.93, 1.07]	[0.90, 1.01]	[0.91, 1.04]
Metal roof	1.55***	2.17***	1.44	0.93
	**[1.13, 2.12]**	**[1.58, 2.99]**	[0.92, 2.27]	[0.54, 1.58]
Improved drinking water access	0.45***	0.67*	1.23	1.08
	**[0.32, 0.61]**	**[0.44, 1.02]**	[0.84, 1.80]	[0.67, 1.75]
Treats drinking water at home	1.84***	0.86	1.12	1.47
	**[1.28, 2.65]**	[0.58, 1.26]	[0.60, 2.10]	[0.69, 3.13]
Durable latrine floor	0.72	0.94	1.67**	1.39
	[0.43, 1.18]	[0.6, 1.46]	**[1.08, 2.60]**	[0.85, 2.28]
Clean latrine floor	1.32**	1.18	1.02	1.04
	**[1.01, 1.71]**	[0.91, 1.54]	[0.49, 2.09]	[0.48, 2.29]
Village-level latrine use >75%	1.35	1.68**	2.21***	1.87**
	[0.85, 2.14]	**[1.06, 2.65]**	**[1.41, 3.45]**	**[1.09, 3.22]**

*Abbreviations*: NL, natural leader; OR, odds ratio; SNNP, Southern Nations, Nationalities, and Peoples. Odds ratios were modeled using an unadjusted logistic regression (one explanatory variable) and a multivariable logistic regression (all explanatory variables). Square brackets are 95% confidence intervals for the odds ratios. All variables are binary except household size. All analysis accounts for unequal selection probabilities, non-response rates, and village-clustering. Coefficients with p < 0.1 are in bold.

* *p* < 0.1.

** *p* < 0.05.

*** *p* < 0.01.

## 4 Discussion

We resurveyed households from villages in Ethiopia and Ghana one year after implementation of four CLTS interventions ended to assess if sanitation outcomes were sustained. In the year after implementation ended, reductions in open defecation were sustained for three of four CLTS interventions evaluated. Only one intervention saw reversion back to open defecation. The average reversion rate in this study was lower than seen in a previous Plan International study in Ethiopia, Kenya, Uganda, and Sierra Leone, in which 13% of households reverted to open defecation in the two-plus years since CLTS had ended (Tyndale-Biscoe et al., 2013). However, these reversion rates are not necessarily inconsistent with our own, as they used a longer follow-up period, and reversion to open defecation may not be a linear process. They also may have overestimated reversion, as they assumed that “open defecation free” status as verified by local government was an accurate measure, which may not be true.

Our finding of no reversion in behavior for three interventions is striking. The majority of latrines in study villages were unimproved, which in this case means their floors and slabs were made of low durability local materials. CLTS often results in low durability latrines made of local materials, which is frequently cited as causing reversion to open defecation (Cavill et al., 2014). Better market access may help prevent this – in Ghana, where study villages were wealthier and closer to markets, 81% of latrines had intact superstructures offering complete privacy, whereas in Ethiopia only 6% did. Many households in this study had latrines fall into disrepair or collapse in the year following implementation – 45% in Ethiopia and 6% in Ghana – but they repaired or rebuilt them in the same year. The high repair rates likely indicate a social norm around latrine use, given that the influence of external facilitators and the incentive of pending ODF certification were gone. While households were clearly committed to continued latrine use (demonstrated by latrine repair rates), a 45% annual latrine disrepair/collapse rate seems likely to discourage households and eventually push them back to open defecation.

The impact of CLTS and subsequent sustained latrine use varied more by region than by intervention, indicating that context may be as or more important than the implementation approach in determining effectiveness. In both Ethiopia and Ghana, the interventions were most effective and the impacts most sustained in remote villages, which were poorer, had higher baseline open defecation, lower prior exposure to WaSH projects, and indicators of potential social cohesion (such as being smaller and having lived together longer). These align with the CLTS Handbook, which describes similar types of villages as where CLTS is most likely to succeed (Kar and Chambers, 2008). These also align with the preliminary findings of a WaterAid study in Nigeria, which found that CLTS was most effective in small, rural, homogenous communities (Abramovsky et al., 2016).

It is reasonable that the interventions we evaluated led to more sustainable outcomes than has been seen previously. Three of the four interventions incorporated training to build capacity within communities. Building local capacity and engaging local leaders has been reported as an enabling factor for other WaSH behaviors, such as sustainability of household-water treatment and storage (HWTS) practices (Ojomo et al., 2015). Trained local actors influencing the behavior of their peers fits with diffusion theory, in which, among other factors, peer-communication and opinion leaders influence the adoption of a new behavior (Rogers, 2003). A more thorough discussion of how training local actors can influence sanitation behaviors is found in our previous Ghana evaluation.

### 4.1 Limitations

This study had a number of limitations. The Ghana interventions did not include a baseline survey, so baseline open defecation levels were estimated based on self-reported latrine age at the midline survey. There was no do-nothing control group in either Ethiopia or Ghana. The timepoints for surveying were different in Ethiopia (0, 12, and 24 months) and Ghana (18 and 30 months). The outcome variable was the primary place of defecation at a household-level, which is not as accurate as measuring individual-level behavior and including secondary practices, and may underestimate open defecation. In order to be conservative, households reporting using latrines that surveyors were not permitted to observe, or that were observed to be unusable (unstable, collapsed, or full), were categorized as open defecation. This may overestimate open defecation (though may have a balancing effect on the previous limitation).

## 5 Conclusion

This study provides evidence of sustained behavior change one year after CLTS implementation ended in Ethiopia and Ghana. Our previously published evaluations of the four CLTS interventions focused on how local actors and different settings changed CLTS effectiveness. From Ethiopia, we concluded that teachers should be trained to support CLTS facilitation, and that CLTS was not an appropriate intervention where baseline open defecation was low (Crocker et al., 2016b). From Ghana, we concluded that training natural leaders can drive deep reductions in open defecation when targeted to socially cohesive villages (Crocker et al., 2016a). From both, we concluded that CLTS should not be a standalone strategy for addressing sanitation, since it was not effective in all settings, and it resulted in low durability latrines.

These conclusions are strengthened by the new evidence on sustained sanitation outcomes presented here. In Ethiopia, the less effective teacher-facilitated CLTS caught up with HEW-facilitated CLTS in the second year. In Ghana, the impact of training natural leaders was sustained. Across both Ethiopia and Ghana, behavior change only occurred in regions characterized by smaller, more remote villages with high baseline open defecation and indicators of social cohesion, which strongly reinforces our previous recommendation to target CLTS to certain settings. This study provides new evidence that CLTS outcomes can be sustained in the short term when the interventions include training and capacity building for local actors. Studying longer term sustainability of CLTS outcomes remains a research priority, as does evaluating CLTS in combination with supply-side interventions and interventions to address household resource constraints.
