# Supplementary material for: Sustainability of community-led total sanitation outcomes: Evidence from Ethiopia and Ghana
Source: Int J Hyg Environ Health. 2017 May;220(3):551–7. doi: 10.1016/j.ijheh.2017.02.011 (PMC5475437; doi:10.1016/j.ijheh.2017.02.011)
Supplement: Supplementary file 1 [file mmc1.docx]

**Appendix A**

**Table S1. Implementation timeline for two CLTS interventions in Ethiopia.**

| **Period** |  | **HEW facilitated CLTS** | **Teacher facilitated CLTS** |
| --- | --- | --- | --- |
| Sep 2012 |  | District orientation | District orientation |
| Oct 2012 |  | Pre-triggering | Pre-triggering |
| Nov 2012 |  | HEW and kebele leader training | Teacher and kebele leader training |
| Nov 2012 - Jan 2013 |  | Triggering | Triggering |
| Dec 2012 - May 2013 |  | Follow-up | Follow-up |
| Mar 2013 |  | HEW and kebele leader review meeting | Teacher review meeting |
| Jun - Nov 2013 |  | ODF certification and celebrations | ODF certification and celebrations |
| Sep 2013 |  | Natural leader training | Natural leader training |

**Table S2. Implementation timeline for two CLTS interventions in Ghana.**

| **Period** |  | **NGO facilitated CLTS + NL training** | **NGO facilitated CLTS** |
| --- | --- | --- | --- |
| Nov 2012 - Jan 2013 |  | District orientation | District orientation |
| Oct 2012 - Jan 2013 |  | Pre-triggering | Pre-triggering |
| Dec 2012 - Mar 2013 |  | Triggering | Triggering |
| Mar 2013 |  | - | Natural leader training |
| Jan 2013 - Mar 2014 |  | Follow-up | Follow-up |
| May 2013 |  | - | Natural leader review meeting |
| Sep 2013 |  | - | Natural leader review meeting |
| Sep - Dec 2013 |  | ODF certification and celebrations | ODF certification and celebrations |
| Dec 2013 |  | - | Natural leader refresher training |
| Feb 2014 |  | - | Natural leader review meeting |

**Table S3. ODF certification status after 12 months of CLTS for four interventions in Ethiopia and Ghana.**

| **Country** | **Intervention** | **ODF certification after 12 months of CLTS (as of December 2013)^a^** |
| --- | --- | --- |
| Ethiopia | Teacher CLTS | 2/4 kebeles (58/111 villages) |
|  | HEW CLTS | 2/2 kebeles (54/54 villages) |
| Ghana | NGO CLTS | 10/29 villages |
|  | NGO CLTS + NL training | 5/29 villages |
| ^a^ODF certification was conducted by district governments. The research team did not observe or validate ODF status. | | |

Table S4. Census and survey sampling counts for villages in four CLTS interventions in Ethiopia and Ghana.

| **Country** | **Intervention** | **Villages sampled** | **Households** | **Surveyed households** | | |
| --- | --- | --- | --- | --- | --- | --- |
|  |  |  |  | Baseline | Midline | Endline |
| Ethiopia | HEW CLTS | 32/54 | 1,624 | 975 | 989 | 971 |
|  | Teacher CLTS | 43/111 | 3,838 | 1,207 | 1,274 | 1,266 |
| Ghana | NGO CLTS | 29/29 | 3,443 | - | 875 | 816 |
|  | NGO CLTS + NL training | 29/29 | 3,312 | - | 833 | 778 |
| Abbreviations: HEW, health extension worker; NGO, non-governmental organization; NL, natural leader. | | | | | | |

**Figure S1. Sanitation practice for households receiving CLTS interventions in Ethiopia and Ghana.** Communal latrines are those that can be used by anyone. Shared latrines are those used by multiple households but not open to the public. Private latrines are used only by one household. Baseline was just before the interventions began. Midline was after CLTS interventions ended (12-months post-baseline in Ethiopia, and 18-months post-baseline in Ghana). Endline was 1-year after midline. Baseline surveys were not used in Ghana.
